# Acute effects of different conditioning activities on running performance of sprinters

**DOI:** 10.1186/s40064-016-2860-7

**Published:** 2016-07-28

**Authors:** Takaya Yoshimoto, Yohei Takai, Hiroaki Kanehisa

**Affiliations:** 1 Japan Institute of Sports Sciences, Kita, Tokyo Japan; 2 National Institute of Fitness and Sports in Kanoya, Kanoya, Kagoshima Japan

**Keywords:** Quickness, Speed, Plyometric exercises, Sprint performance

## Abstract

This study investigated acute effects of different conditioning activities on sprint performance of collegiate sprinters using a randomized, crossover design. Male sprinters (N = 10; 20.1 ± 0.6 years; 174.6 ± 4.4 cm; 66.7 ± 3.5 kg; 100-m race personal best time, 11.46 ± 0.57 s; means ± SDs) performed two 60-m sprints and one of three treatments within the same day, with an interval of 2 days between the treatments. The baseline sprint was followed by one of three different conditioning activities: mini-hurdles, bounding jumps, or a free sprint. Participants then performed the post treatment sprint. In the mini-hurdle drill, the participants ran over 10 × 10 mini-hurdles (height 22 cm) as fast as possible. In the bounding jump drill, the participants performed three 60-m bounding jumps as explosively and far as possible, with 3 min intervals between trials. In the free-sprint conditioning activity, the participants performed a 60-m maximal sprint twice, with a 5 min interval between sprints. Sprint kinematics in the baseline and post treatment sprints were recorded using a high-speed camera (300 Hz). Using these films, sprint time, running velocity, step length, and step frequency were analyzed over 10 m intervals. The results of ANOVAs indicated that the mini-hurdle drill increased the maximal sprint velocity (3.2 %) and maximal step frequency (3.3 %); the other conditioning activities had no such effects. Step length did not change after any of the conditioning activities. These results suggest that conditioning activities with mini-hurdles, which require movements with a high step frequency, acutely enhances velocity during sprinting, possibly as a result of increasing step frequency.

## Background

Sprinters in track and field events must run a given distance with maximal effort. A 100 m race mainly consists of three main phases: the acceleration, maximal speed, and speed maintenance phases (Ross et al. 2001). Maćkala (2007) has demonstrated that the average sprinters (100 m race time of 11.18 s) accelerate until approximately 40 m, and attain maximal speed in the phase from 40 to 60 m. The maximal speed during a race is correlated with the race time (Mackala 2007) and depends on the preceding gain in speed during the acceleration phase (van Ingen Schenau et al. 1994). Therefore, the ability of the sprinter to accelerate during a limited time is an important factor in achieving high performance in 100-m racing events (van Ingen Schenau et al. 1994). Running velocity is theoretically the product of both step frequency and step length. However, an increase in one parameter typically leads to a decrease in the other (Hunter et al. 2004; Debaere et al. 2013). Debaere et al. (2013) demonstrated that step length was significantly correlated with maximal running velocity during sprinting. Hunter et al. (2004) also pointed out that inter-subject differences in maximal running velocity depended on step length. However, they demonstrated that intra-subject variation in race time was caused by changes in step frequency and not by those in step length. This suggests that variation in race time within a sprinter may be attributable to changes in step frequency.

It is also known that sprint performance can be acutely enhanced by using resistance (Bevan et al. 2010) or plyometric exercises (Turner et al. 2015). These forceful and/or explosive activities, being conducted prior to the main exercises, are called as conditioning activities (Fukutani et al. 2014). Turner et al. (2015) reported that plyometric exercises as conditioning activities produced an acute improvement in sprint velocity during the acceleration phase of a sprint in well-trained men. To the best of our knowledge, no studies have systematically examined the effects of conditioning activities on the sprint performance of sprinters. Crewther et al. (2011) have demonstrated that implementing a single set of 3-repetition maximum back squat exercises increased countermovement jumps, but did not change 5- and 10-m sprint times. They suggested that such enhancements have a certain degree of task specificity, such that effects of conditioning activities are greater for the performances of athletic events in which movement patterns are similar to those of the conditioning activity. If this is so, plyometric exercises used as a conditioning activity may acutely induce step length-dependent enhancement in sprint performance, because step length is related to the explosive force/power-generating capacity of the lower extremities (Kale et al. 2009).

On the other hand, step frequency-dependent changes in sprint performance might be observed after exercises that involve movements with high step frequency, because of the similarity in the task demands. If acute enhancement of sprint performance has a certain degree of task specificity, actual sprinting as a conditioning activity would be appropriate for enhancing the performance of acute sprinting.

This study was designed to clarify the effect of conditioning activities on the performance of sprinters. We hypothesized that the acute enhancement of sprint performance following conditioning activities would result if these activities were task-specific.

## Methods

This study was approved by the ethical committee of the National Institute of Fitness and Sports in Kanoya and was consistent with its requirements for human experimentation. Prior to the experiment, all subjects were informed of the experimental procedures and possible risks of the measurements. Written informed consent was obtained from each subject. The purpose and hypothesis of the study were not explained to the subjects until termination of measurements, to exclude any potential bias that might affect the results.

### Participants

Male sprinters (N = 10; age 20.1 ± 0.6 years; height 174.6 ± 4.4 cm; weight 66.7 ± 3.5 kg; 100-m personal best time, 11.46 ± 0.57 s; means ± SDs) participated in this study. They were free of cardiovascular, metabolic, and immunologic disorders and orthopedic abnormalities, and were not using any medications that affected their muscle functions. All participants had been competing in short distance track and field events (100, 200, and 400 m) for more than 4 years. All participated in specific training programs for sprinters with a coach for at least 3 h/day on 5 days/week.

### Experimental procedure

**Fig. 1:**
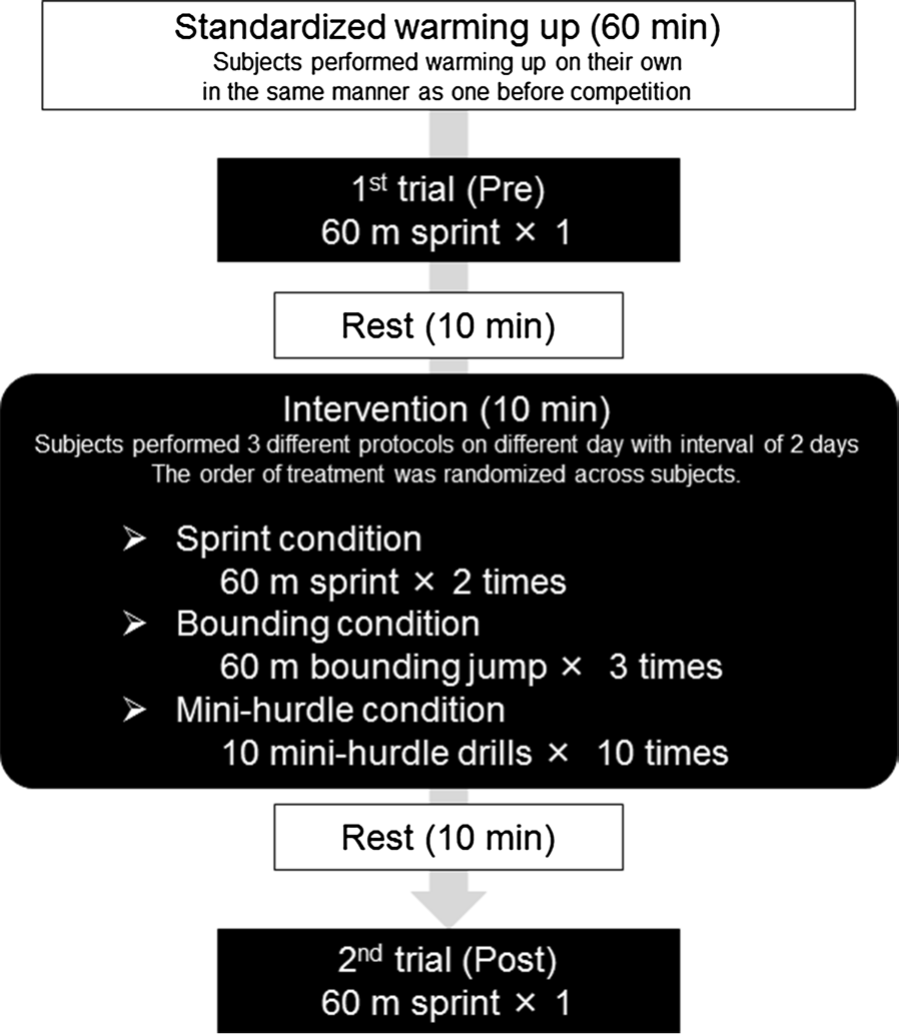
Experimental design

We compared sprint performance at baseline with sprint performance after conducting different conditioning activities, one of which involved actual sprinting. Participants were assessed in three experimental sessions with an interval of 2 days between each session. In each session, they performed a different conditioning activity. Figure 1 presents the study design. One hour prior to the baseline assessment (described below), participants first performed their usual pre-competition warm-up exercises, which were not specified but left to their discretion. After completing warm-up exercises and before the treatment, all participants performed a baseline 60-m maximal sprint on a synthetic surface track wearing spiked shoes (Pre). They took a 10-min rest, and then performed one of the three conditioning activities for 10 min with a coach’s encouragement. In the 60-m free sprint, participants sprinted twice for over 60 meters, at maximal effort, with an interval of 5 min between sprints, starting from a crouched position. In the 60-m bounding jump, which required explosive and horizontal jumping movements, participants jumped three times, each time as far as possible horizontally, using alternating legs and with their arms swinging, with an interval of 3 min between jumps. In the mini-hurdle drill, which involved movements with high step frequency, participants ran three times, each time as fast as possible, over 10 mini-hurdles (height 22 cm and spaced 90 cm apart) with an interval of 3 min between trials. The order of the treatments over days was randomized across participants. After completing each treatment, participants ran 60 m again with maximal effort (Post).

### 60-m sprint performance test

Running kinematics during the 60-m sprint before and after each treatment was recorded using a high-speed camera (EX-F1, CASIO, Japan) at a sampling frequency of 300 frames/s (shutter speed, 1/1600 s). The camera was set 30 m away from the lateral edge of the running course, such that it panned across the runway. Lines were drawn every 10 m on the lane, and markers were set between the centers of each line and the camera. The running time, running velocity, and the number of steps were averaged over every 10 m using the films. Step length was determined by dividing running velocity by step frequency, and was averaged over every 10 m. The highest running velocity among the 6 phases was labeled the maximal running velocity, and step frequency and step length in the corresponding phase were adopted as the maximal step frequency and step length, respectively. In addition, the mean values of running velocity, step length, and step frequency were calculated by averaging the corresponding values across the phases. In this article, the term “step frequency” is defined as the number of steps per second in half the running cycle, that is, from foot contact to the next contact of the opposite foot. The term “step length” is defined as the distance from foot contact to the next contact of the opposite foot.

### Statistical analysis

Independent variables in the analysis were maximal and mean running velocity, step length and step frequency. Descriptive statistics are presented as means and SDs. A one way repeated measures analysis of variance (ANOVA) with a Bonferroni post hoc test was conducted to test the significance of differences among the three conditions for each of the independent variables at Pre. Intra-class correlation coefficients (ICCs) and confidence intervals (CIs) were calculated for the corresponding values. Coefficients of variation (CVs) for sprint time, maximal running velocity, mean running velocity, step length, and step frequency for 60 m at Pre were calculated by dividing SDs by means across all sessions, and multiplying the values by 100 (%). A two-way repeated measures ANOVA (2 times: Pre, Post × 6 phases of running distance) was conducted to test the significance of differences for each of the independent variables. A two-way repeated measures ANOVA (3 conditions × 2 times: Pre, Post) was conducted to test the significance of differences between Pre and Post for 60 m time, maximal running velocity, step length, and step frequency for each of the independent variables. When a significant interaction was found, a simple main effects test was used for post hoc comparison. Effect size (ES) was calculated for each condition as (means value at Post − means value at Pre)/SDs at Pre. ES was classified as trivial (ES < 0.2), small (0.2 < ES < 0.5), moderate (0.5 < ES < 0.8), or large (ES > 0.8). Statistical significance was set at p < 0.05. All data analyses were conducted using the statistical software (SPSS 19.0 for Windows, IBM, Japan).

## Results

There were no significant differences among the three conditions for sprint time, maximal and mean running velocity, step length, and step frequency during the 60-m sprint at Pre. The ICCs (CIs) of the values at Pre were 0.925 (0.805–0.979) for sprint time, 0.935 (0.829–0.982) for maximal running velocity, 0.929 (0.814–0.980) for mean running velocity, 0.857 (0.655–0.958) for step length, and 0.858 (0.657–0.958) for step frequency. The CVs of the values at Pre in each condition were 1.1 ± 0.2 % for time, 1.4 ± 0.6 % for maximal running velocity, 1.1 ± 0.2 % for mean running velocity, 1.4 ± 0.4 % for step frequency, and 1.3 ± 0.5 % for step length.

In the free-sprint condition, all effect sizes were trivial, and there were no significant main effects of time and no significant interactions for running velocity (F = 0.07, *df* = 1, ES: 0.00–0.09), step length (F = 0.3, *df* = 1, ES: 0.01–0.08), or step frequency (F = 0.02, *df* = 1, ES: 0.00–0.09). In the bounding jump condition, effect sizes were trivial or small and no significant effects were found on running velocity (F = 0.2, *df* = 1, ES: 0.01–0.47), step length (F = 0.9, *df* = 1, ES: 0.05–0.36), or step frequency (F = 1.2, *df* = 1, ES: 0.00–0.27). In the mini-hurdle condition, there were significant main effects of time on running velocity (F = 12.6, *df* = 1, ES: 0.19–0.63, *p* < 0.05) and step frequency (F = 20.3, *df* = 1, ES: 0.19–0.50, p < 0.05). A significant interaction between time and phase was also found on running velocity (F = 0.4, *df* = 1, *p* < 0.05). A simple main effects test revealed that running velocity increased significantly increased in the phases of 0–10, 20–30, and 40–50 m phases after the treatment, with small to moderate effect sizes. At 50–60 m, running velocity tended to increase after the treatment (*p* = 0.060), with a small effect size. Step length did not change significantly after the treatment (F = 0.9, *df* = 1, ES: 0.07–0.41), with effect sizes trivial or small.

Table 1 presents descriptive statistics on the sprint time across 60 m and maximal values of running velocity, step length, and step frequency for the 6 phases before and after each treatment. A significant interaction between condition and time was found on sprint time (F = 8.8, *df* = 2, ES: 0.30, *p* < 0.05), maximal running velocity (F = 7.7, *df* = 2, ES: 0.58, *p* < 0.05), and step frequency (F = 3.9, *df* = 2, ES: 0.58, *p* < 0.05), with moderate effect sizes. A simple main effects test indicated that maximal running velocity and step frequency in the mini-hurdle condition increased by 3.2 and 3.3 %, respectively. No significant main or interaction effects of time were found on step length. The initial values of the measured variables at Pre were not significantly related to relative changes in the independent variables from Pre to Post (r = −0.015 to −0.612, *p* > 0.05). The 10-m split time of each treatment is shown in Table 2. The 10 m split time decreased in the mini-hurdle condition, but did not change with the other conditioning activities.

**Table 1:** Descriptive statistics of 60 m sprint time, maximal running velocity, step length and step frequency across 60 m

	Free sprint condition			Bounding jump condition			Mini-hurdle condition		
	Pre	Post	%	Pre	Post	%	Pre	Post	%
60 m sprint time (s)	7.84 ± 0.33	7.85 ± 0.32	0.1	7.83 ± 0.30	7.80 ± 0.27	−0.5	7.90 ± 0.35	7.79 ± 0.35^a^	−1.5
Running velocity (m/s)	9.26 ± 0.57	9.27 ± 0.49	0.1	9.26 ± 0.52	9.33 ± 0.41	0.8	9.16 ± 0.50	9.45 ± 0.58^a^	3.2
Step length (m)	2.14 ± 0.13	2.15 ± 0.09	0.3	2.17 ± 0.15	2.16 ± 0.12	−0.1	2.14 ± 0.13	2.14 ± 0.12^a^	0.0
Step frequency steps (s)	4.33 ± 0.28	4.32 ± 0.18	0.0	4.28 ± 0.25	4.32 ± 0.23	1.0	4.29 ± 0.23	4.42 ± 0.20^a^	3.3

The values are expressed as means and SDs

^a^Significant change from Pre to Post

**Table 2:** Descriptive statistics of the 10 m split time in the three conditions

	0–10 m	10–20 m	20–30 m	30–40 m	40–50 m	50–60 m
Free sprint						
Pre	2.19 ± 0.09	1.21 ± 0.04	1.15 ± 0.05	1.11 ± 0.05	1.09 ± 0.07	1.10 ± 0.06
Post	2.19 ± 0.07	1.21 ± 0.04	1.16 ± 0.05	1.12 ± 0.06	1.09 ± 0.06	1.10 ± 0.07
Bounding jump						
Pre	2.20 ± 0.08	1.20 ± 0.04	1.15 ± 0.04	1.10 ± 0.04	1.09 ± 0.06	1.10 ± 0.06
Post	2.16 ± 0.07	1.21 ± 0.04	1.15 ± 0.04	1.11 ± 0.05	1.08 ± 0.05	1.09 ± 0.05
Mini-hurdle^a^						
Pre	2.22 ± 0.08	1.20 ± 0.05	1.15 ± 0.05	1.12 ± 0.06	1.10 ± 0.06	1.11 ± 0.07
Post	2.17 ± 0.10	1.22 ± 0.04	1.13 ± 0.05	1.10 ± 0.05	1.07 ± 0.07	1.10 ± 0.07

The unit is in seconds

The values are expressed as means and SDs

^a^Significant change from Pre to Post across all phases

## Discussion

The conditioning activity using mini-hurdles, which required movements with a high step frequency, acutely enhanced running velocity in the acceleration and maximal speed phases of the 60-m, maximal intensity sprint, by increasing the step frequency. The free sprint and the bounding jump conditions did not induce significant changes in the measured parameters of sprint performance. In the mini-hurdle condition, the relative change in maximal running velocity (3.2 %) corresponded to that of step frequency (3.3 %), suggesting that the observed gain in running velocity after the mini-hurdles as a conditioning activity depended mainly on the increase of step frequency. The magnitude of the increase was greater for running velocity and step frequency than for CVs of running velocity (1.1 %) and step frequency (1.4 %) at Pre. These results partially support our hypothesis and indicated that the conditioning activity using mini-hurdles, which involved fast movements, facilitated maximal running velocity, which was based on the increase in step frequency.

It is known that there is an inverse relationship between step length and step frequency (Debaere et al. 2013). Thus, an increase in step frequency may attenuate step length. In the current study, however, no significant changes in step length occurred after mini-hurdles were used as a conditioning activity. This suggests that this conditioning activity did not have a negative influence on the step length, although it was associated with an increase in step frequency. Hunter et al. (2004) who compared running kinematics on the first fastest race time to those on the third fastest race time within a sprinter, reported that race times were affected by step frequency, rather than by step length. This suggests that step frequency is more changeable than step length from race to race within a sprinter, and this change influences race time.

Crewther et al. (2011) demonstrated that a 3-repetition-maximum squat exercise as a conditioning activity acutely improved vertical jump height from baseline, but it did not affect sprint time. They suggested that the gain in athletic performance after an active squatting exercise depends on movement specificity. Applied to the current study, this suggests that the 60-m free sprint as a conditioning activity would be an appropriate preparation for a 60-m test sprint. However, our findings did not support this idea. There was no significant change in sprint performance scores following either a 60-m free sprint, or a bounding jump as conditioning activities. Only the conditioning activity focused on movements with a high step frequency was effective in enhancing acute sprint performance. However, we cannot conclude that the observed increase in step frequency was a unique effect of the mini-hurdle drill adopted here, because there are other ways to induced movements with a high step frequency, such as using ropes or cones. Further study is needed to investigate these possibilities.

There were no significant changes in sprint performance after the bounding jump protocol. This refuted our hypothesis that plyometric exercise as a conditioning activity would result in step length-dependent enhancement, because of the similarity in the required force/power-generating capacity of the lower extremities required to perform this exercise. Previous findings on the effects of plyometric exercises as conditioning activities on sprint performance remain equivocal. As cited earlier, variation in step length cannot be a factor in explaining variation in the race time within an individual sprinter (Hunter et al. 2004). The conditioning activities used in this study did not induce a significant change in step length. Thus it may be difficult to induce acute step length-dependent enhancements of sprint performance through conditioning activities.

In this study, the inter-subject variation in personal best records for 100 m events was large (11.46 ± 0.57 s). The relative changes in sprint performance scores after conditioning activities were not significantly related to the initial values at Pre, suggesting that enhancement of sprint performance following the prescribed conditioning activity may be independent of the sprint abilities of individuals.

Several limitations of this study should be noted. It has been suggested that the acute enhancement of athletic performance following resistance or plyometric exercises as a conditioning activity can be attributed to an increase in muscle temperature, nerve conductivity, metabolic reaction rate, the phosphorylation of myosin regulatory light chains, and recruitment of higher order motor units (Bishop 2003; Hodgson et al. 2005). In addition, it is known that the magnitude of the acute performance enhancement is influenced by the intensity of exercise used as a conditioning activity (Fukutani et al. 2014; Hirayama 2014). Prior to the baseline measurement, participants performed their usual 1-h warm-up exercises, those they perform before competition races. Thus, participants were adequately warmed up for the subsequent sprint. However, we did not match the total workload in each of the three conditioning activities, because they were designed on the basis of standardized drills generally used by sprinters. The total distance covered in the mini-hurdle task was shorter than that in the other tasks. Thus, we cannot rule out effects of total workload differences among the three conditioning activities on measured parameters. A pilot study indicated that blood lactate concentration after the prescribed conditioning activities tended to be lower in the mini-hurdle condition than in the bounding jump and free sprint conditions (Yoshimoto et al. 2014). This implies that the anaerobic energy requirement may have been greater in the bounding jump and free sprint conditions than in the mini-hurdle condition. Fukutani et al. (2014) suggested that the effect of conditioning contraction on the subsequent maximal voluntary concentric torque would be influenced by muscle fatigue induced by the conditioning contraction. Ross et al. (2001) also reported that neural fatigue, exhibited as suprasupinal failure, segmental afferent inhibition, depression of motor neuron excitability, and loss of excitation at branch points, is a potential limiting factor during maximal sprint exercise and for a period of time following it. Considering these findings, the absence of acute change in sprint performance after the free sprint and bounding jump treatments may have been attributable to neuromuscular fatigue, which would be depend upon the intensity and/or volume of the two treatments.

## Practical application

The conditioning activity using mini-hurdles, which required movements with a high step frequency, acutely enhanced running velocity in the acceleration and maximal speed phases of the 60-m sprint by increasing the step frequency. Sprinters usually perform adequate warm-ups prior to competition. Considering the current findings, strength and conditioning coaches should include mini-hurdle exercises in their warm-up programs as a conditioning activity to improve sprint performance.

## Conclusion

Conditioning activities using mini-hurdles, which involve movement with a high step frequency, acutely enhances sprint velocity by increasing step frequency. The present findings suggest that the use of bounding jumps and free sprinting as conditioning activities require careful consideration because of the possible occurrence of neuromuscular fatigue, depending on their intensity and/or volume.
